# Biodegradable Nanocomposite of ZnS(Mn) Quantum Dots Immobilized Graphene Oxide for Bioimaging Applications

**DOI:** 10.7150/ntno.87536

**Published:** 2024-01-21

**Authors:** Pavithra Kurungottu, Midhun Ben Thomas, Mahesh M Lalitha, Prathiksha Ganesh, Divya Prakash Gnanadhas, Dipshika Chakravortty, Ashok M Raichur, Rajendra Kurapati

**Affiliations:** 1 School of Chemistry, Indian Institute of Science Education and Research Thiruvananthapuram, Thiruvananthapuram, India.; 2 Department of Materials Engineering, Indian Institute of Science, Bangalore, India.; 3 Department of Microbiology and Cell Biology, Indian Institute of Science, Bangalore, India.; 4 Department of Bioengineering, School of Chemical and Biotechnology, SASTRA Deemed to be University, Thanjavur, Tamilnadu., India.

## Abstract

Developing a biocompatible and biodegradable graphene-based fluorescent nanoprobe with the ability to visualize live cells could be interesting for intracellular imaging and monitoring the efficiency of chemotherapy. Herein, we report a biodegradable and biocompatible hybrid fluorescent graphene oxide (GO)-ZnS(Mn) composite synthesized via *in situ* growth of ZnS(Mn) quantum dots (QDs) on the surface of GO in the aqueous medium. The prepared 'GO-ZnS(Mn)' composite was characterized by X-ray diffraction (XRD), X-ray photoelectron spectroscopy (XPS), thermogravimetric analysis (TGA) and high-resolution transmission electron microscopy (HR-TEM) along with selected area electron diffraction (SAED). Further, the fluorescence properties of the GO-ZnS(Mn) composite were studied using fluorescence emission spectroscopy. The composite material exhibited a strong and broad visible light fluorescence from 500 to 600 nm by excitation with 365 nm (UV) light. The cytotoxic experiments of folic acid (FA) conjugated GO-ZnS(Mn) using MTT [(3-[4,5-dimethylthiazol-2-yl]-2,5 diphenyl tetrazolium bromide)] assay revealed that the composite had excellent biocompatibility even at higher concentrations up to 200 µg/mL in HeLa cell lines. Next, the bioimaging experiments carried out using confocal fluorescence laser scanning microscopy (CLSM) revealed that GO-ZnS(Mn) composite was taken up by the HeLa cells effectively within 12 h of incubation via receptor (folate) mediated endocytosis with strong fluorescence throughout the cell surface. Finally, the biodegradability of GO-ZnS(Mn) composite was studied by treating it with human myeloperoxidase enzyme (hMPO) isolated from the primary immune cells, neutrophils, which is important to understand the *in vivo* fate of GO-Zns(Mn). The HR-TEM and Raman analyses confirmed the biodegradation of GO-ZnS(Mn) within 15 h of hMPO treatment. Thus, the biodegradable GO-ZnS (Mn) composite could be helpful for chemotherapy and bioimaging applications.

## Introduction

Among graphene derivatives, graphene oxide (GO) contains various hydrophilic oxygenated groups (e.g., carboxylic, hydroxyl, epoxide, etc.), rendering excellent dispersibility in the aqueous media, higher biocompatibility and biodegradability over other carbon materials.^1^-^3^ Thus, the properties make GO a potential candidate for drug/gene delivery, antibacterial, tissue engineering, photothermal and photodynamic therapies.^4^, ^5^ Among the applications of GO, bioimaging is a major concern since obtaining strong luminescent GO is not facile with tunable emission, which helps in theranostics applications.^6^, ^7^ To overcome this limitation, semiconductor quantum dots (QDs) were employed to be immobilized on the surface of the GO, thereby making the GO-QDs composite a tunable photoluminescent material for bioimaging applications.^7^ In general, semiconductor QDs such as CdS, CdSe, CdTe, etc., have gained huge attention as fluorescent bio-probes for sensing and bioimaging applications.^8^-^12^ However, most of the QDs suffered from being cytotoxic and poor water dispersibility due to the presence of heavy metals and their synthesis using nonpolar solvents.^13^ By immobilizing these QDs onto the GO surface, the water dispersibility and biocompatibility of QDs were also enhanced significantly. For example, Cd-based QDs (CdTe and CdS) were incorporated into the surface of GO, and the resulting GO composites showed excellent photoluminescence and enhanced cellular uptake.^14^-^16^ Furthermore, Hu et al. reported the 11-mercaptoundecanoic acid (MUA)-capped CdSe/ZnS QDs onto reduced graphene oxide (rGO) for imaging human carcinoma (HeLa) cells.^17^ However, these QDs were considered to be carcinogenic due to the presence of Cd. Later, zinc-doped AgInS_2_ (AIZS) QDs-GO fluorescent composite was developed for bioimaging applications with tunable photoluminescent properties.^7^ Although AgInS_2_ is biocompatible over Cd-based QDs, these QDs found to be genotoxic and affect mitochondrial dysfunction.^18^ *In vivo* bioimaging of cancer tissues was also reported using GO-AgInS_2_ composite, where the cytotoxicity was increased beyond 0.8 µg/mL.^13^ Next, CuInS_2_/ZnS QDs toxicity levels and cancer therapy effectiveness were improved by integrating them into the rGO surface utilizing PEGylated liposome as the linking reagent.^19^

Among QDs, ZnS was found to be stable, biocompatible and affordable for bioimaging applications.^20^, ^21^ Further, doping of Mn^2+^ ions into ZnS reduced its inherent quenching behavior and improved the fluorescence efficiency near the visible region, including high biocompatibility for targeted cancer imaging.^22^ These ZnS QDs were immobilized onto the graphene surface using hydrothermal co-precipitation, exhibiting the strong fluorescence.^23^ As ZnS(Mn) exhibits phosphorescence emission (~590 nm), the interference from biological tissues can be avoided as phosphorescence, making it an ideal candidate for biomedical applications.^24^, ^25^^,^ However, most of the QDs were immobilized on the graphene surface via hydrothermal assisted co-precipitation above 150 ^°^C resulting reduced graphene oxide (rGO), which is less favored over GO for biomedical applications due to higher water dispersibility and biocompatibility of GO. Similarly, immobilization of ZnS(Mn)-rGO (via hydrothermally, 170 ^°^C for 5 h) was reported for cancer theranostics applications.^26^ Therefore, obtaining ZnS(Mn) incorporated into the GO sheets could be more desirable for bioimaging and theranostics applications.

On the other hand, the biodegradability of the nanomaterials will decide their fate in the clinical translation.^3^ In this regard, the biodegradable luminescent nanomaterials-based fluorescent probes are more fascinating for better clinical translation compared to classical non-degradable and heavy metal-based QDs. For example, porous iron oxide doped silica particles showed excellent fluorescent properties and biodegradability for *in vivo* bioimaging.^27^ In this respect, the biodegradability of GO-QDs composites will be more attractive to understand the *in vivo* persistency and body clearance. 3 To date, no study is reporting the biodegradability of GO-QDs composite, especially in the bioimaging and theranostic applications. Herein, we synthesized strong fluorescent biodegradable and biocompatible GO-ZnS(Mn) composite for bioimaging applications. The monodispersed ZnS(Mn) were immobilized on the surface of GO via the *in-situ* growth method using microwave irradiation in an aqueous suspension. In addition, GO was conjugated with folic acid for specific interaction with folate receptors over-expressed on the cancer cell lines. Next, we studied the *in vitro* cytotoxicity of GO-ZnS(Mn) composite using HeLa cells and carried out bioimaging applications of the GO-ZnS(Mn) for visualizing the HeLa cells. Finally, GO-ZnS(Mn) composite biodegradability was studied by treating with human myeloperoxidase (hMPO) isolated from the innate immune cells, neutrophils. The biodegradability and bioimaging studies suggest the applicability of GO-ZnS(Mn) composite for *in vivo* theranostics or bioimaging applications.

## Materials and Methods

High purity graphite powder with an average grain size of 45 µm was purchased from Sigma-Aldrich. Sulphuric acid (H_2_SO_4_), hydrochloric acid (HCl), sodium nitrite (NaNO_2_), sodium hydroxide (NaOH), sodium nitrate (NaNO_3_), zinc acetate [Zn(CH_3_COO)_2_], manganese sulfate (MnSO_4_), and sodium sulphide (Na_2_S) were purchased from SRL India. Enzyme hMPO derived from human neutrophil, hydrogen peroxide (H_2_O_2_, 30% aqueous solution), NaCl, NaH_2_PO_4_·2H_2_O, Na_2_HPO_4_·2H_2_O and DTPA (diethylenetriamine pentaacetate) were purchased from Sigma-Aldrich. The water used in all the experiments was obtained from the Milli-Q system with a resistivity greater than 18 MΩ. 4',6-diamidino-2-phenylindole (DAPI), propidium iodide (PI) solution, glutaraldehyde, phosphate buffered saline (PBS) tablets were all purchased from Sigma-Aldrich and used without any further purification. For pH adjustments, 0.1 M HCl or 0.1 M NaOH solutions prepared in DI water were used.

### Synthesis of graphene oxide (GO) and conjugation with folic acid (FA)

GO was synthesized by the modified Hummer's method, similar to our previous works.^28^, ^29^^,^ Next, as synthesized, 50 mL of exfoliated GO was sonicated for 2 h, followed by the addition of an equal volume of 4 µM of EDC [1-ethyl-3-(-3-dimethylaminopropyl) carbodiimide hydrochloride] and 10 mM of NHS (*N*-hydroxysuccinimide) in the ratio 25:1 (v/v). This was gently vortexed for 20 min before adding folic acid (FA) in the ratio 20:1 (v/v) and the resulting sample was kept overnight under gentle stirring.

### Synthesis of hybrid GO-ZnS (Mn) composite

After the conjugation of GO with FA, the sample was subjected to treatment with the precursors of zinc sulphide. 50 mL of 0.1 M Zn(CH_3_COO)_2_ solution was added to the GO sample, followed by the addition of 7.5 mL of (15 atomic %) MnSO_4_. It was added dropwise under ultra-sonication and incubated for 30 min. This was followed by a dropwise addition of 50 mL of 0.1 M sodium sulfide under moderate stirring. The sample turned into a dirty white color, which indicated the formation of ZnS doped with manganese, which was then subjected to microwave irradiation (domestic microwave, IFB Model 30BRC3, power output 900 W, 2450 MHz at 80 ^°^C) for 1 min. After centrifugation at 3000 rpm for 5 min at room temperature, the precipitates were washed with distilled water five times and redispersed in double distilled water.

To ascertain the formation of doped ZnS(Mn) QDs, the sample's fluorescence was observed with a UV lamp with an excitation wavelength of 365 nm. The colloidal sample exhibited orange red emission, which was characteristic of ZnS doped with Mn^2+^ and it was observed that optimal doping of manganese was obtained when the pH of the sample was ~ 6.

### Characterization methods

The exfoliated GO, GO-ZnS(Mn) and ZnS(Mn) were characterized by transmission electron microscopy (TEM) (FEI Tecnai TF30 HR-TEM) for which the samples were drop-casted on a 300-mesh carbon-coated copper grid (Ted Pella, Inc) and dried overnight at room temperature in a desiccator. The atomic force microscopy (AFM) images of GO were taken with the Nanosurf Easyscan 2 after the samples were placed on mica sheets and dried at room temperature. Powder X-ray diffraction (P-XRD) analyses were performed using Panalytical powder XRD (CuKα, λ = 1.5406 Å, 40 kV, 40 mA) in 2θ range of 5 - 80° and with a scan rate of 1.8° per min. The zeta potential of the GO sheets was studied using the Zetasizer Nano ZS with 655 nm laser, the samples were diluted with DI water to obtain the well-dispersed samples before keeping them in the electrophoretic cell. Each measurement was taken as the average of three separate readings. Photophysical studies are done using a quartz cuvette of 10 mm path length and the absorption spectra were recorded using Shimadzu UV-3600 Vis-NIR spectrophotometer and emission spectra were recorded on spectrofluorometer (Horiba Jobin Yvon- Fluorolog 3). The GO-ZnS(Mn) was dispersed in DI water at 0.5 mg/mL for the fluorescence studies. Next, the Raman spectroscopy analyses of GO, GO-ZnS(Mn) and ZnS(Mn) were recorded using LabRaM HR (UV) spectrophotometer with 514 nm laser excitation (2 mW) power at room temperature. X-ray photoelectron spectroscopy (XPS) analyses of the samples was performed using Omicron Nanotech XPS in CAE analyzer mode with a 1253.6 eV MgKα excitation source with a pass energy of 50 eV. The thermogravimetric analysis (TGA) of the samples was performed using Hitachi STA 200 thermal analysis system from room temperature to 1000 ^ᴏ^C.

### Biodegradation of GO-ZnS(Mn) composite using hMPO in the presence of H_2_O_2_

The procedure to study the biodegradation of GO-ZnS(Mn) composite was adapted from our previous work mimicking the *in vivo* reaction of hMPO in the presence of H_2_O_2_.^30^ Briefly, 0.032 mg of GO-ZnS(Mn) composite was resuspended in 200 μL of PBS containing 140 mM NaCl and 100 μM DTPA. To which 12.5 μL of 100 μg/μL hMPO was added, followed by the addition of 200 μM H_2_O_2_ for every hour up to 15 h in total and hMPO was refreshed every 5 h. The whole experiment was conducted in an incubator set at 37 ^°^C. Similar to the experimental sample, the control samples including only GO-ZnS(Mn) treated with H_2_O_2_ in the absence of hMPO, ZnS(Mn) with H_2_O_2_ in the absence of hMPO, GO-ZnS(Mn) only in PBS were also tested similarly for 15 h. Aliquots containing 10 μL of samples were collected at 0 and 15 h and stored at -20 ^°^C for further characterization.

### MTT assay

Cytotoxicity of the GO-ZnS(Mn) composite was analyzed *in vitro* by MTT assay in HeLa cell lines.^31^ HeLa cell lines were maintained in Dulbecco's Modified Eagle's Medium (DMEM; Sigma) supplemented with 10% fetal calf serum (Sigma). All cells were held at 37 °C and 5% CO_2_. Cells were seeded on a 96-well plate at a cell density of 5x10^4^ cells/mL. After 8 h, various GO-ZnS(Mn) concentrations were added and incubated for 12 h. MTT dye (20 µL of 5 mg/mL) was added to each well and kept for 4 h at 37 °C. The percentage of cell viability was determined at 570 nm relative to non-treated cells.

### Bioimaging of HeLa cells

Confocal images were taken using a Carl Zeiss LSM Confocal scanning system (Carl Zeiss, Jena, Germany) equipped with a 100x oil immersion objective with a numerical aperture of 1.4. HeLa cells (1x10^5^ cells/well) were seeded in a sterile glass coverslip in a 24-well plate and incubated for 8 h. Then the cells were incubated with FA-GO-ZnS(Mn) for different durations at 5 µg/mL concentration, repeatedly washed and stained with 1 µg/mL of DAPI for nuclear staining. The cells were fixed with 4% paraformaldehyde and visualized under confocal microscopy.

## Results and Discussion

### Synthesis and characterization of GO-ZnS(Mn) composite

**Scheme 1:**
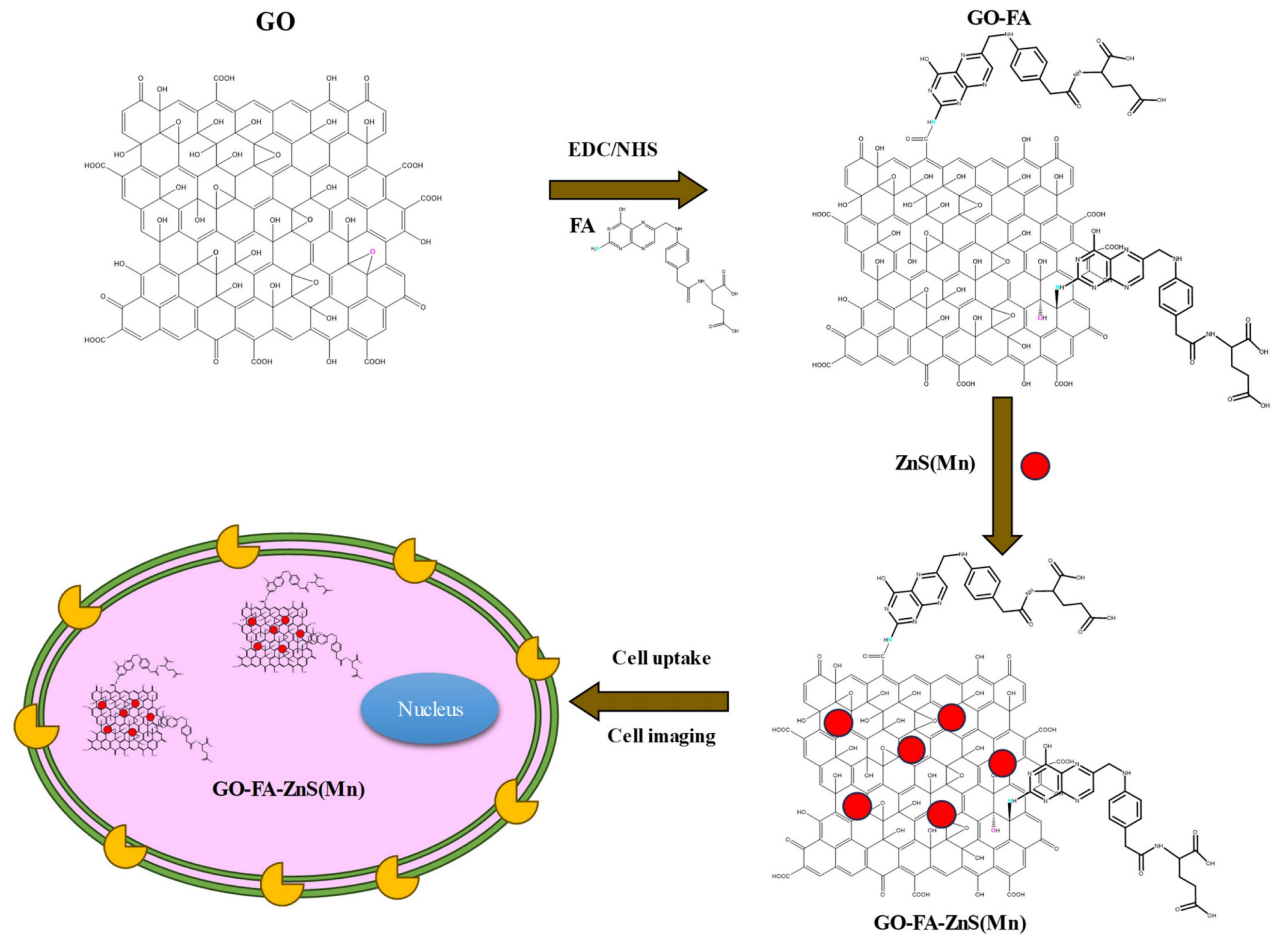
Illustration of synthesis procedure for FA conjugated GO-ZnS(Mn) composite, specific cellular uptake, and bioimaging using the fluorescence of GO-ZnS(Mn) composite.

**Figure 1:**
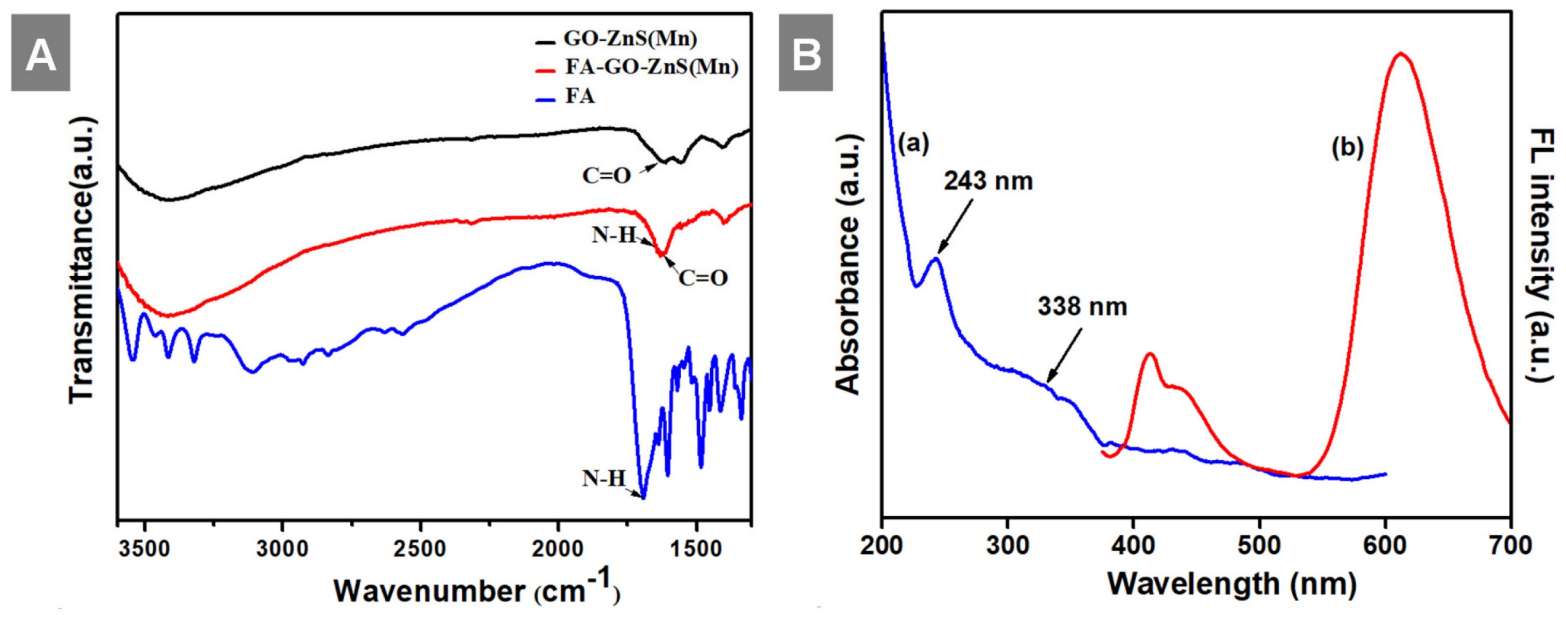
(A) FT-IR spectra of GO-ZnS(Mn), FA-GO-ZnS(Mn), and FA alone; and (B) UV-vis spectrum (a) and the fluorescence spectrum (b) of GO-ZnS(Mn) composite, respectively.

At first, highly aqueous dispersible GO was obtained via a modified Hummers' method and characterized using microscopic and spectroscopic techniques similar to our previous works.^28^, ^29^ The exfoliated GO sheets were observed under TEM as displayed in Figure S1 (Supp Info). The thickness of exfoliated GO sheets was found to be 1 to 2 nm as measured using AFM, which confirmed the existence of mono or double layer GO as shown in Figure S2.^32^ The high colloidal stability of GO aqueous suspension was supported by its high negative zeta potential (- 43.0 mV). In the next step, folic acid (FA) was conjugated to GO via EDC/NHS coupling as shown in Scheme 1 and followed by *in-situ* growth of ZnS(Mn) QDs by treating with resulting GO-FA composite in the aqueous suspension. The coupling of FA with GO was confirmed by FTIR spectroscopy for GO-FA-ZnS(Mn) as shown in Figure 1A, where FA exhibited a characteristic combinational vibrational bands at 1612 cm^-1^ corresponded to the N-H bending and C-N stretching vibrations (amide II mode).^26^ Further, the amide bond formation between FA and GO was confirmed by observing the characteristic band at 1554 cm^-1^ corresponding to the C=O stretching vibration of amide I mode.^26^ The FA conjugation can also be possible via the opening of epoxide groups present on the surface of GO by treating with FA (Scheme 1) as reported earlier.^33^

Next, UV-vis studies with GO and GO-ZnS(Mn) composite were performed to confirm the *in situ* growth of ZnS(Mn) QDs on the surface of GO sheets. The characteristic absorption peak of GO was observed at 230 nm (Supp Info, Figure S3) and this peak was red-shifted to 243 nm for GO-ZnS(Mn) due to the interaction of QDs with GO, as reported earlier.^15^ Further, the broad absorption peak centered at ~338 nm is due to the presence of ZnS(Mn) QDs, confirming the successful growth of QDs on the surface of GO.^15^, ^34^ The fluorescence (FL) of GO-ZnS(Mn) QDs was confirmed by fluorescence spectroscopy as shown in Figure 1B. The peak at ~460 nm is present in GO-ZnS(Mn) composites arising from the defect states of ZnS nanocrystals involved in radiative recombination.^35^ There was also a broad peak around ~600 nm in both the spectra of GO-ZnS(Mn) corresponding to the ^4^T_1_ → ^6^A_1_ d-d transition of Mn^2+^ confirming the successful doping of Mn in ZnS QDs.^36^ Later, FL quantum yield of the GO-ZnS(Mn) composite was determined according to the following equation;



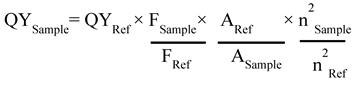



Where F is the area under the spectrum, A is the absorbance and n is the refractive index.^37^ Coumarin-153 dye dissolved in ethanol was used as the reference (Ref) with QY= 0.554,^38^ and the quantum yield of GO-ZnS(Mn) composite is 0.0003; this value is in good agreement with the previously reported QY for ZnS(Mn) QDs.^37^

**Figure 2:**
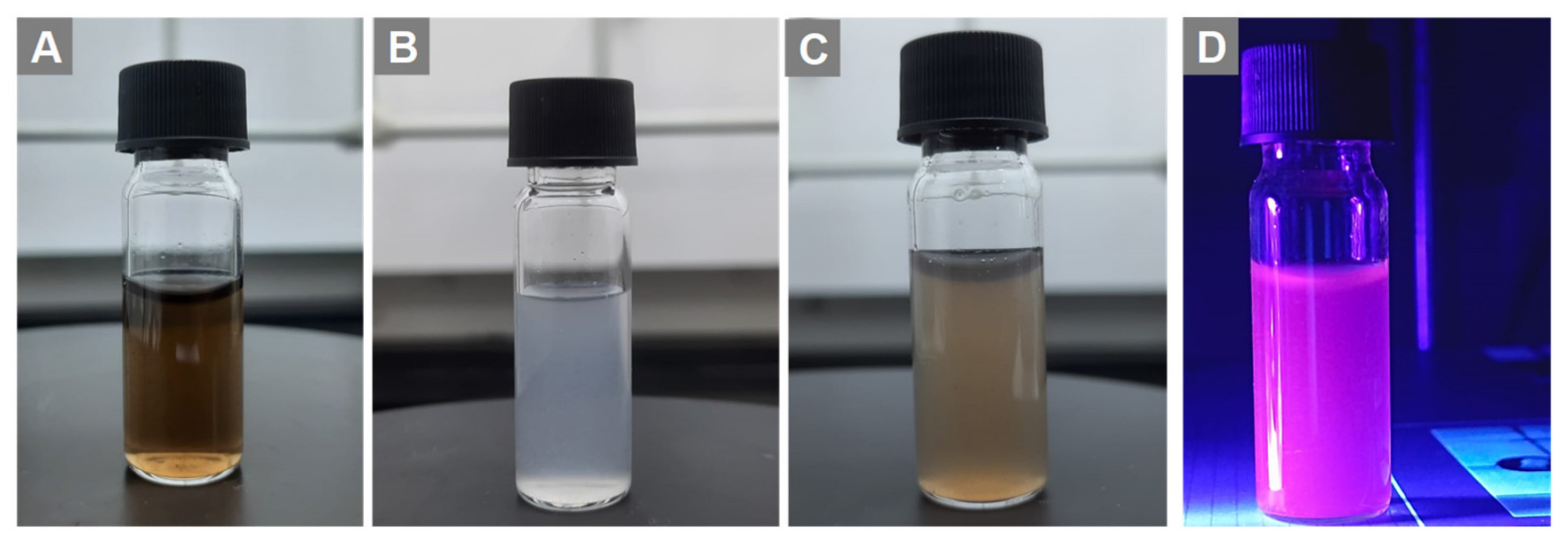
Digital photos of (A) the GO aqueous suspension, (B) aqueous ZnS(Mn) alone, (C) composite of GO-ZnS(Mn) and (D) aqueous GO-ZnS(Mn) composite under UV light exposure at a wavelength of 365 nm, respectively.

We tested the fluorescence properties of GO-ZnS(Mn) composite aqueous suspension under UV light at 365 nm wavelength (Figure 2), where the dark pink color was clearly observed for GO-ZnS(Mn) composite under UV light (Figure 2D). In addition, there was a clear difference in the visible colors of GO (Figure 2A), ZnS(Mn) (Figure 2B**)** and GO-ZnS(Mn) (Figure 2C) in the absence of UV light, which supports the formation of the GO-ZnS(Mn) composite. Notably, the 'physical mixture' of ZnS(Mn) QDs with GO [GO+ZnS(Mn)] did not show such pink color fluorescence (data not shown here) unlike *in situ* grown GO-ZnS(Mn) composite, which is attributed to the fluorescence quenching phenomena of graphene based materials.^39^ Thereby indicating the significance of *in-situ* growth method to obtain high luminescent GO-ZnS(Mn) composite.

**Figure 3:**
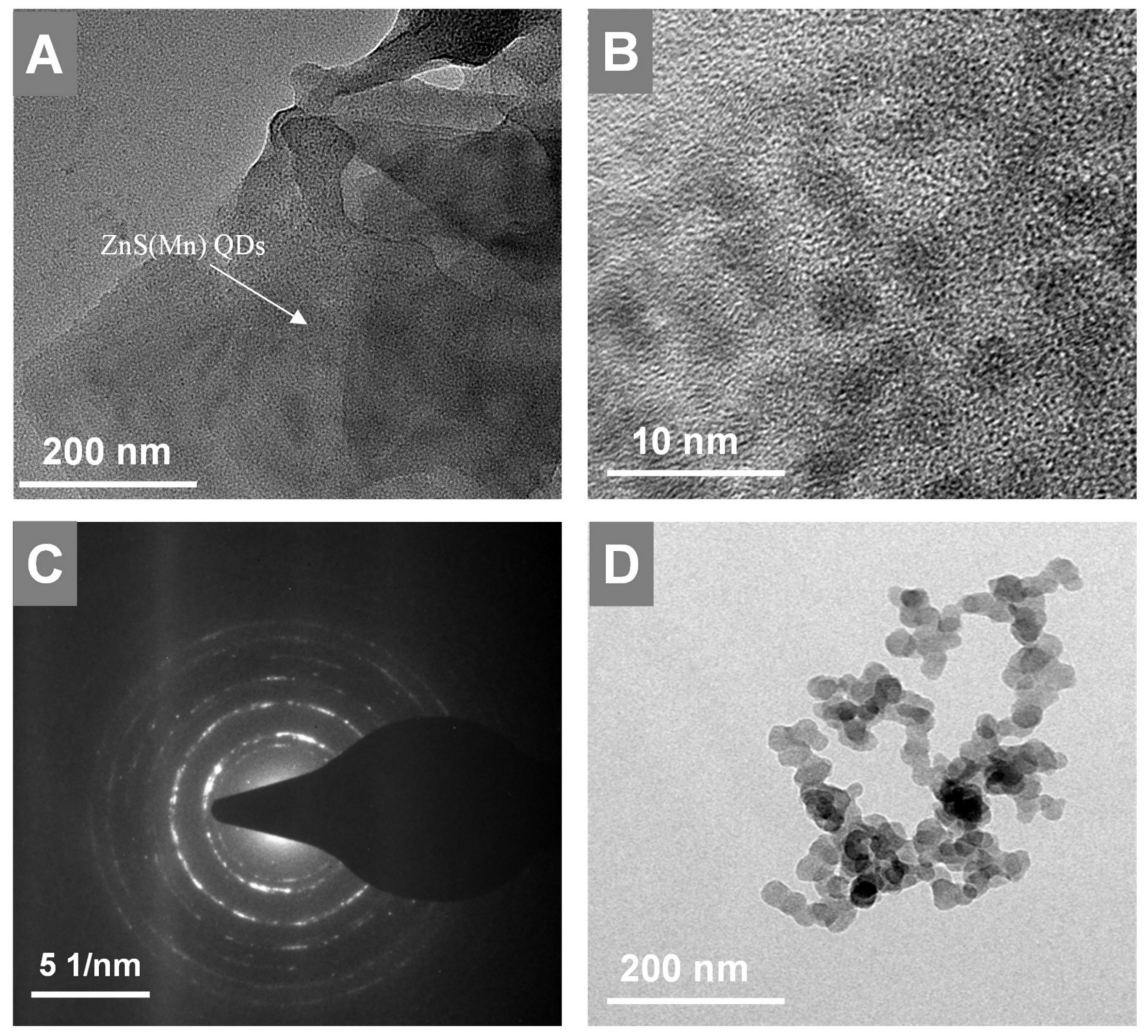
HR-TEM images of GO-ZnS(Mn) at low and high magnification shown in A and B, respectively, (C) represents the SAED pattern of the GO-ZnS(Mn) composite, (D) HR-TEM image of ZnS(Mn) particles alone without immobilization on the GO.

Next, the *in-situ* grown GO-ZnS(Mn) composite was analyzed using HR-TEM as shown in Figure 3. The high dispersible ZnS(Mn) QDs were observed on the surface of GO (Figure 3A-B, Supp Info S4) confirming the successful immobilization of QDs, where the size of ZnS(Mn) QDs was ~3 to 4 nm. The electrostatic interaction between the carboxylic acid groups, hydroxy groups present on the graphitic lattice of GO, and Zn^2+^ ions is the main reason for the effective immobilization of ZnS(Mn) QDs over the surface of GO without any aggregation.^15^, ^26^ The diffused rings observed in the selected area diffraction (SAED) pattern confirmed the polycrystalline nature of GO-ZnS(Mn) as shown in Figure 3C. In addition, we used energy dispersive X-ray spectroscopy (EDS) attached to TEM to check the elemental composition of GO-ZnS(Mn) hybrid sheets, as shown in Figure S5 (see Supp info). The peaks corresponding to C, O, Mn, S and Zn were clearly observed in the EDS spectra, which strongly provided evidence for the formation of ZnS(Mn) on the surface of GO via *in-situ* growth mechanism.

**Figure 4:**
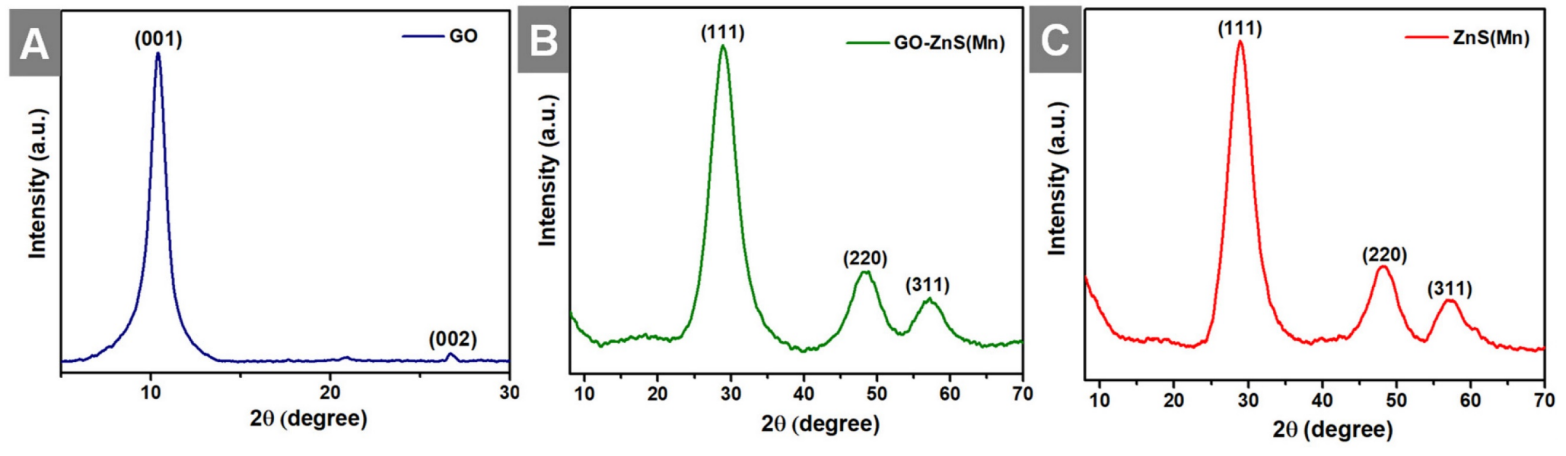
Shows the XRD patterns of GO (A), GO-ZnS(Mn) (B) and ZnS(Mn) (C), respectively.

Further, X-ray diffraction (XRD) analyses were performed for all the samples, including GO, GO-ZnS(Mn) and ZnS(Mn) QDs from 2ϴ = 10 to 80^o^ as shown in Figure 4. The XRD pattern of GO showed an intense peak at a low-angle scattering area around 10° corresponding to the (0 0 1) plane of exfoliated GO,^40^ where a much weak intense peak at 26.62^o^ attributed to unexfoliated graphite (Figure 4A). The XRD results of composite GO-ZnS(Mn) showed the peaks at 28.5^o^, 48.4^o^ and 56.5^o^ correspond to (1 1 1), (2 2 0) as well as (3 1 1) planes of cubic ZnS (zinc blende) structure (Figure 4B), which are in good agreement with the standard card (JCPDS no. 5-0566).^41^ The similar peaks were also observed for ZnS(Mn) QDs, as shown in Figure 4C thereby confirming the presence of ZnS(Mn) QDs on the GO surface.^26^ The characteristic peak of GO at 10° was not observed in the GO-ZnS(Mn), which is attributed to the lower diffraction intensity of GO than ZnS QDs as reported earlier.^26^

**Figure 5:**
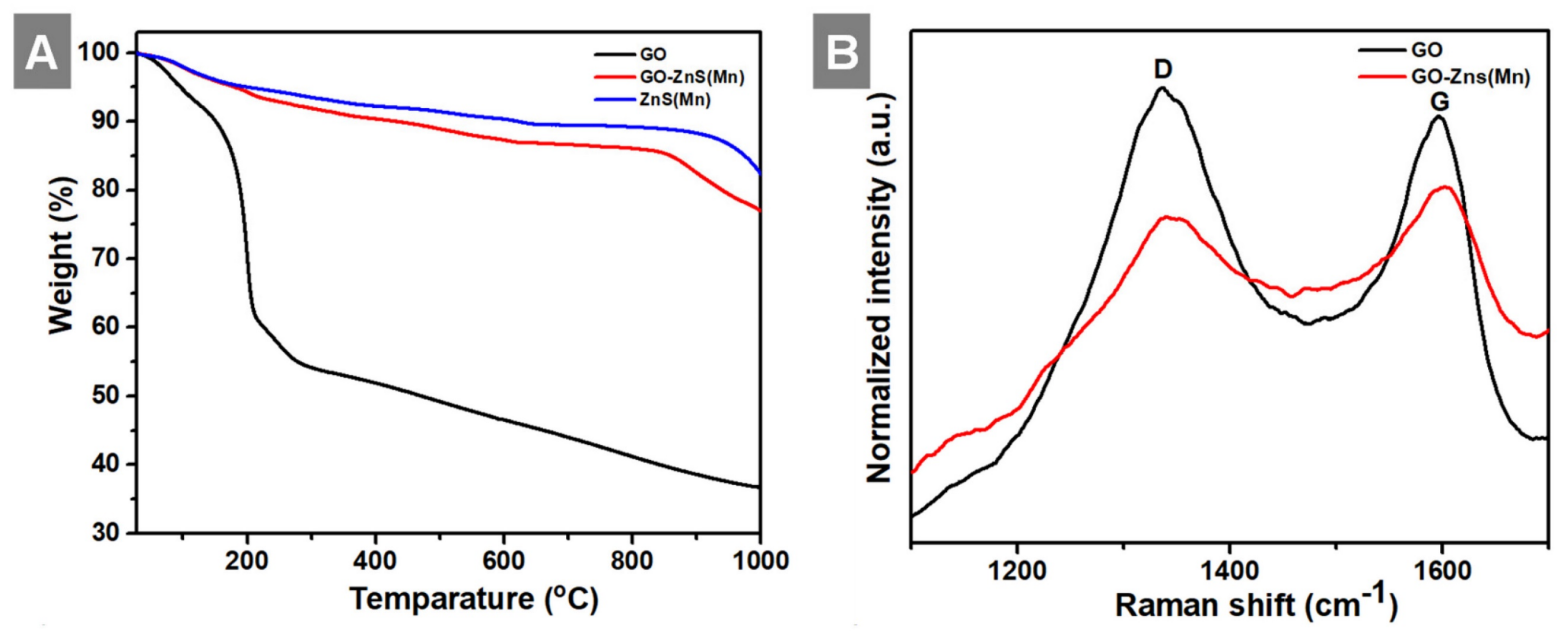
Shows (A) TGA curves of GO, ZnS(Mn) QDs and GO-ZnS(Mn) composite and (B) the Raman spectrum of GO-ZnS(Mn) and GO, respectively.

In the next step, we analyzed the thermal behavior of the GO-ZnS(Mn), ZnS(Mn) composite and GO as shown in Figure 5A. The GO-ZnS(Mn) composite sheets have exhibited higher thermal stability than GO alone. The initial weight loss observed up to 200 ^°^C was ascribed to the desorption of surface-bound and absorbed water molecules from GO and GO-ZnS(Mn).^42^ TGA analysis of GO alone displayed a distinct weight loss of ~35% at ~200°C due to loss of water content and oxygenated groups on the surface of graphene.^43^ Further weight loss above 200 ^°^C is attributed to the liberation of CO_2_ due to the thermal decomposition of GO and destruction of carbon framework up to 700 °C.^44^ At 1000 °C, only 30% mass was left, but the GO-ZnS(Mn) composite had around 75% weight, possibly due to *in-situ* grown ZnS(Mn) QDs on the GO surface. Further, the Raman analysis was performed to reveal whether any partial reduction of GO (rGO) occurred during the microwave treatment (1 min at 85 ^o^ C) during the synthesis of GO-ZnS(Mn). As shown in Figure 5B, the characteristic D and G bands of pure GO appeared at 1337 cm^-1^ and 1596 cm^-1^, respectively. While GO-ZnS(Mn) showed the D and G bands at 1341 cm^-1^ and 1598 cm^-1^ respectively.^26^ The G band corresponds to the sp^2^ hybridized C-C bond stretching of graphene and the D band corresponds to the sp^3^ like defects in the graphitic lattice.^45^ However, there is a slight reduction in the I_D_/I_G_ ratio (1.05) for GO-ZnS(Mn) compared to the GO alone (1.21), suggesting that GO was mildly reduced during the microwave irradiation as reported earlier.^46^

**Figure 6:**
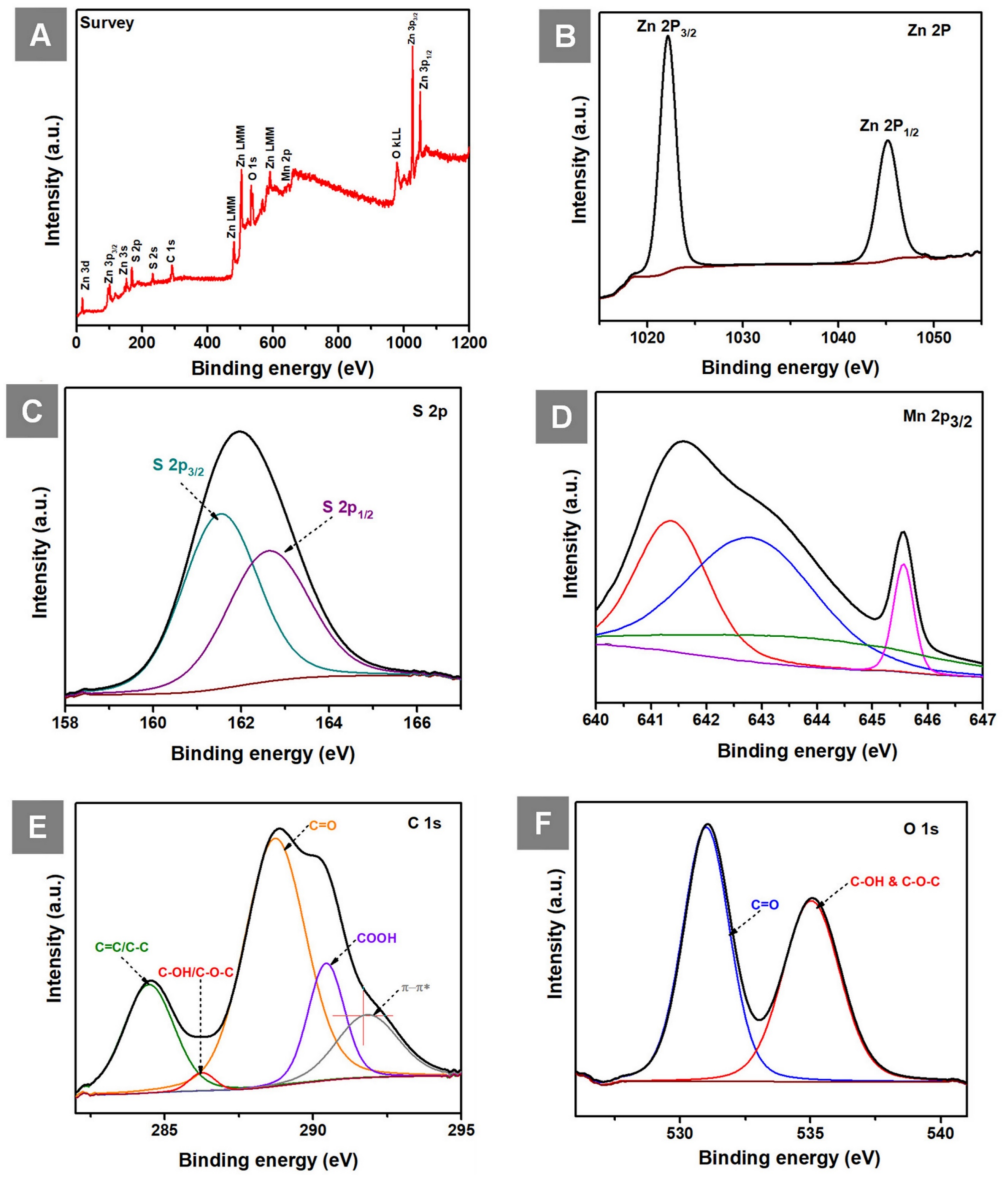
(A) Shows the survey spectrum of GO-ZnS(Mn), and core level XPS spectra of (B) Zn 2p (C) S 2P (D) Mn 2p_3/2_ (E) C 1s and (F) O 1s in GO-ZnS(Mn) composite, respectively.

The XPS survey spectrum of GO-ZnS(Mn) composite shows the presence of Zn, S, Mn, C and O elements as shown in Figure 6A. The high-resolution spectra of Zn 2p (Figure 6B) consist of two peaks one is at 1022.15 (2p_3/2_) and 1045.15 (2p_1/2_) having spin-orbit splitting energy of 23 eV which confirmed the presence of Zn(II). Next, the two peaks centered at 161.46 eV (S 2p_3/2_) and 162.66 eV (S 2p_1/2_) are visible in the deconvolution of the S 2p core level spectrum (Figure 6C), indicating the presence of the bivalent S^2-^ state with its distinctive peak separation of 1.2 eV. Further, the asymmetric Mn 2p_3/2_ core spectrum showed multiple peaks corresponding to Mn^2+^(Figure 6D), which is in different electronic environments, where the presence of a small shake up peak at 645. 52 eV indicated the surface oxidation of Mn.^47^ The XPS core level spectrum of C 1s (Figure 6E) showed peaks at 284.48 eV (C=C and C-C), 286.23 eV (C-OH and C-O-C), 288.73 eV (C=O), 290.43 (COOH) related to the GO and the peak at 291.78 eV related to π- π* shake up.^48^, ^49^ Next, the XPS peak for O 1s (Figure 6F) was splitted into two parts, the first one at 530.99 eV (C=O) and 535.04 eV (C-OH and C-O-C).^50^

### *In vitro* cytotoxicity studies using MTT assay

**Figure 7:**
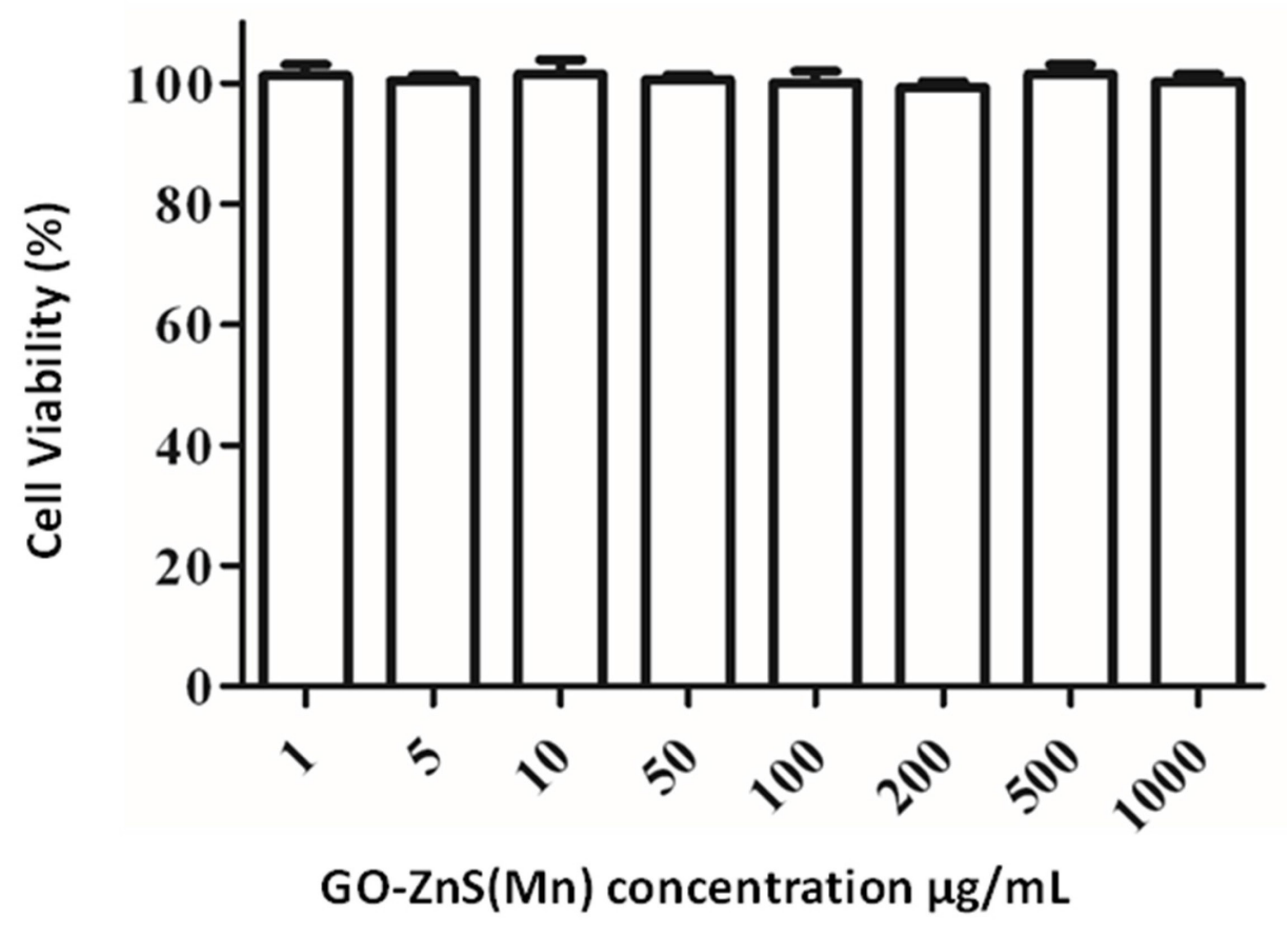
Cell viability assay for cytotoxicity values versus incubation concentration started from 1 µg/mL to 1000 µg/mL of GO-ZnS(Mn) composite in HeLa cells for 12 h.

The biocompatibility of any biomaterial is an important parameter that could judge the fate of any material used in biomedical applications. GO-based materials have shown impressive biocompatibility.^51^ The cytotoxicity of GO-ZnS(Mn) composite was investigated using MTT assay in HeLa cell lines as shown in Figure 7. The cytotoxicity results indicate that concentrations from 1 µg/mL up to 1 mg/mL of GO-ZnS(Mn) did not affect the viability of HeLa cells. However, the GO-ZnS(Mn) composite showed higher cell viability when compared with the CdS QDs (refer to the Supp Info. regarding the synthesis characterization of CdS QDs, Figure S6). The cell viability studies suggested that the CdS QDs slightly affected the cell viability of HeLa cells from 50 to 200 µg/mL concentration (Figure S7, Supp Info) compared to the GO-ZnS(Mn) composite.^22^

### Bioimaging of HeLa cells using GO-ZnS(Mn) composite

**Figure 8:**
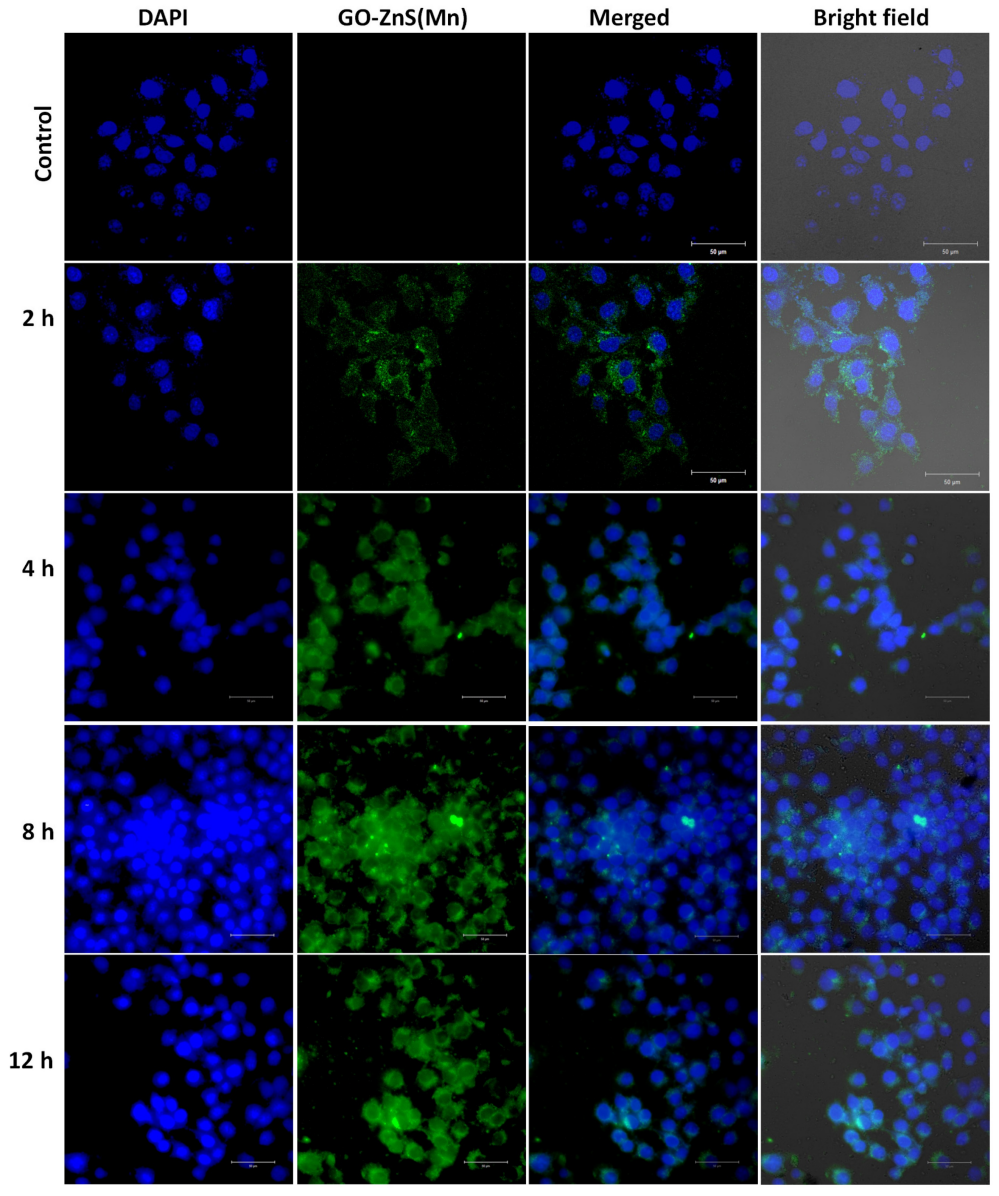
Cellular uptake and bioimaging studies with FA conjugated GO-ZnS(Mn) composite treated with HeLa cells, where the first column shows DAPI stained nuclei, the second column shows green photoluminescence exhibited by the ZnS(Mn) NPs immobilized on the surface of GO, third and fourth column shows the merged and bright field images respectively.

**Figure 9:**
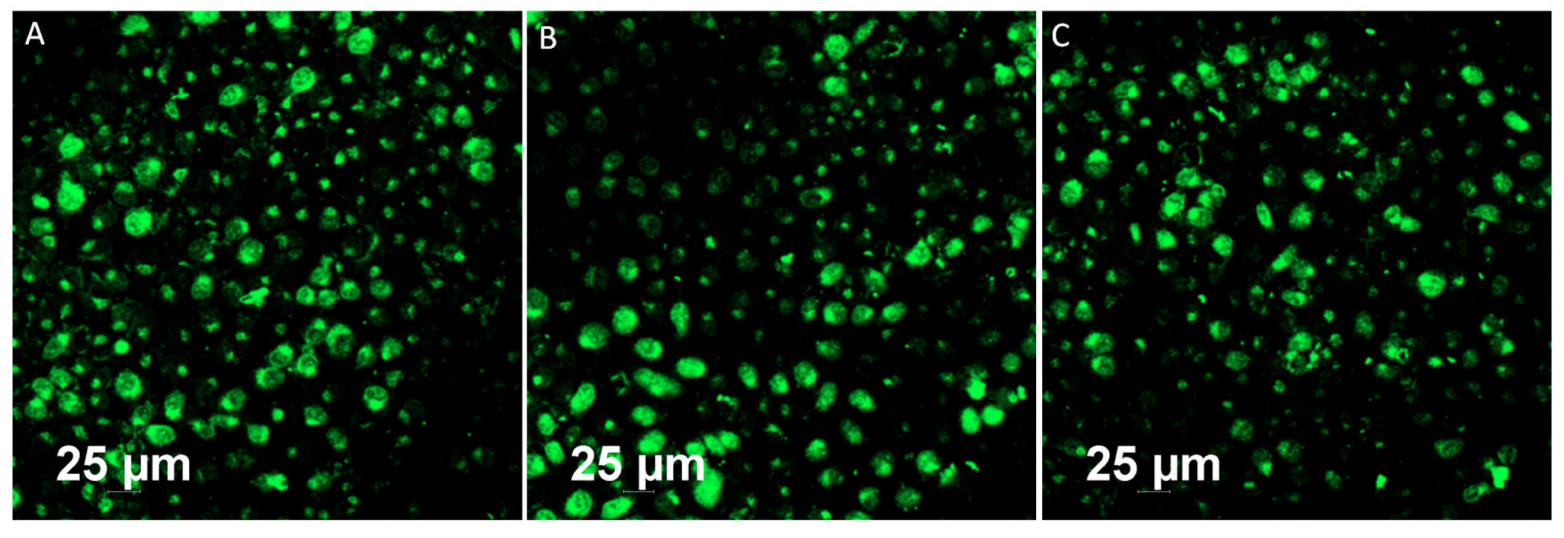
Fluorescent imaging of the HeLa cells after incubating with GO-ZnS(Mn) alone for 12 h without staining the nuclei with the DAPI.

Bioimaging of HeLa cells was studied using the fluorescence properties of GO-ZnS(Mn) composite under a confocal fluorescence microscope, as shown in Figure 8-9. HeLa cells were incubated with GO-ZnS(Mn) particles for different durations to check the effect of the particles on the cells. Most of the particles were observed on the membrane after 1 h of incubation, whereas the entry into the cytoplasm was observed from 2 h. The entry of particles into the cytoplasm increased with incubation time. As shown in Figure 8, the maximum concentration of GO-ZnS(Mn) particles was observed along with strong fluorescence localization in the surrounding cell membrane, including cytoplasm, at 12 h of incubation. To differentiate the bioimaging ability of GO-ZnS(Mn) alone, the HeLa cells were incubated with GO-ZnS(Mn) and the florescent images were taken after 12 h (Figure 9), where the bright green florescent cells were clearly observed confirming the potential of GO-ZnS(Mn) as a bioimaging agent. Thus, we believe this GO-ZnS(Mn) could be effectively used for bioimaging applications, especially in cancer nanotheranostics, where therapy is combined with diagnosis.

### Enzymatic biodegradation of GO-ZnS(Mn) by human myeloperoxidase (hMPO)

**Figure 10:**
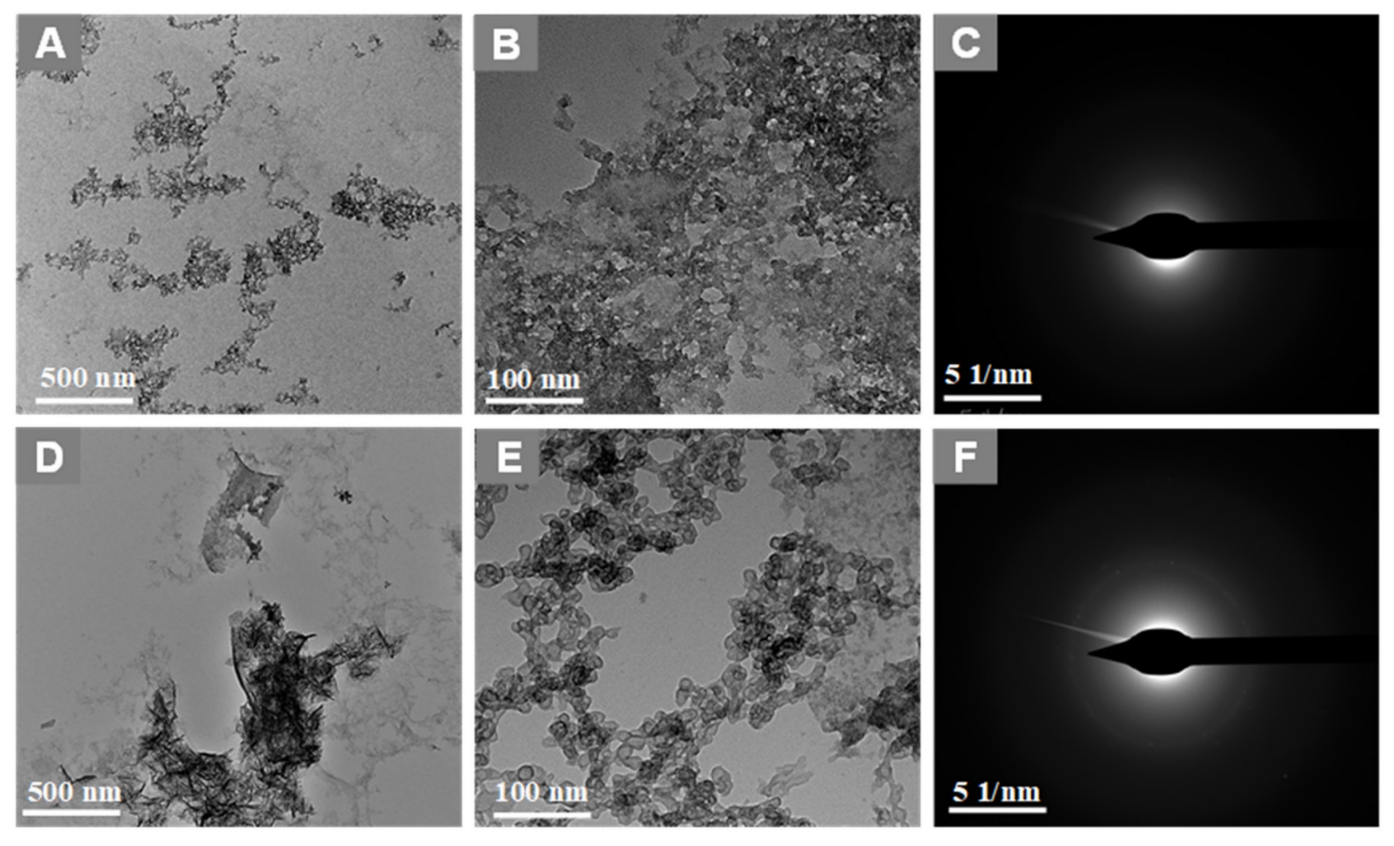
TEM images and SAED pattern of GO-ZnS(Mn) with hMPO+H_2_O_2_ (A-C) and GO-ZnS(Mn) with H_2_O_2_ alone (D-F) after treating for 15 h, respectively.

**Figure 11:**
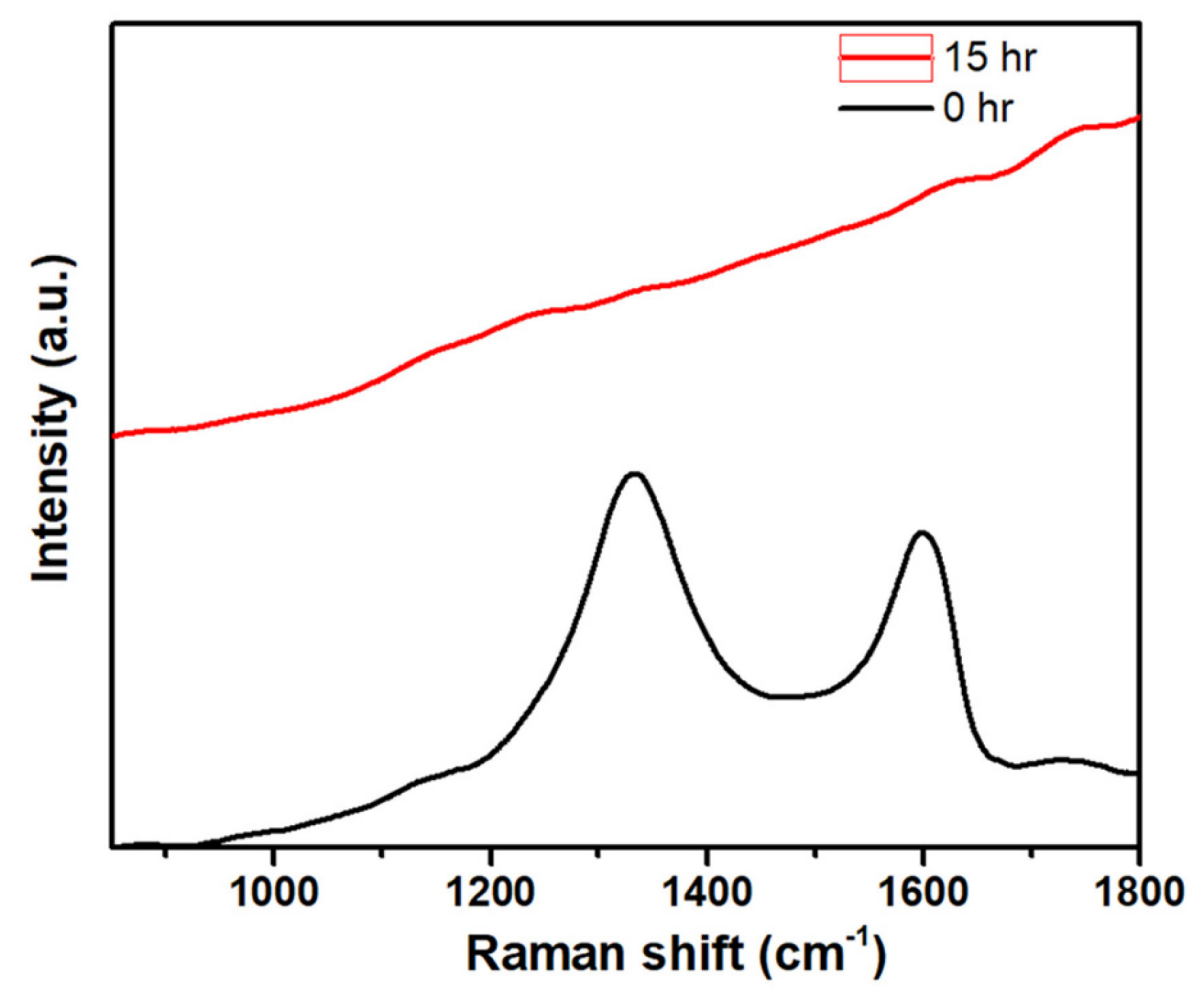
The Raman spectra of GO-ZnS(Mn) composite after treating with hMPO+H_2_O_2_ for 0 and 15 h, respectively, where each spectrum is an average of a minimum of five individual spectra.

Most of the nanomaterials-based biomedical products were not translated to the clinics as they failed in the clinical trials due to their non-degradability.^2^ Therefore, developing biodegradable materials is extremely important for their potential clinic usage. Therefore, understanding the biodegradability of the nanotheranostics materials (bioimaging agents) will be crucial to understanding their *in vivo* fate and biodistribution. In this regard, we investigated the biodegradability of GO-ZnS(Mn) composite for the first time by treating with human myeloperoxidase (hMPO) in the presence of H_2_O_2_. Earlier studies reported that GO could be degraded by the primary immune cells (neutrophils) through the peroxidase activity of hMPO.^2^, ^30^ Hence, it would be interesting to check the biodegradability of GO-ZnS(Mn) composite using such enzymatic action. First, GO-ZnS(Mn) sheets were treated with hMPO and H_2_O_2_ for 15 h and followed the degradation using HR-TEM and Raman spectroscopy. As shown in Figure 10A-B, TEM results suggested that there is a significant degradation of GO-ZnS(Mn) within 15 h of hMPO treatment since the resulting GO-ZnS(Mn) showed highly porous morphology and lost the regular 2D shape compared to the initial GO-ZnS(Mn) (Figure 3A). Further, the SAED pattern (Figure 10C**)** of hMPO-treated GO-ZnS(Mn) displayed an amorphous nature, unlike the polycrystallinity found for initial GO-ZnS(Mn) (Figure 3C), confirming the oxidation/degradation of GO-ZnS(Mn) by hMPO activity. The biodegradability of GO-ZnS(Mn) composite is similar to the degradability of GO alone by hMPO treatment as reported earlier.^3^, ^30^ However, GO-ZnS(Mn) sheets were found to be degraded partially by H_2_O_2_ treatment alone for 15 h as shown in Figure 10D-E, where there are hollow nanostructured particles were found (Figure 10E). Further, SAED analyses confirmed that those hollow nanoparticles are amorphous as they displayed the diffused rings (Figure 10F). Similar kinds of hollow structures were also observed for the ZnS(Mn) QDs after being treated with H_2_O_2_ for 15 h (Supp Info, Figure S8D-F). These nanostructured hollow particles could be due to the release of metal ions and the formation of elemental sulfur after treatment with H_2_O_2,_ as reported earlier^52^. However, the control sample GO-ZnS(Mn) in PBS (Figure S8A-B) did not show any degradation as found in the H_2_O_2_-treated sample, confirming the oxidation of the composite by H_2_O_2_ treatment. Next, the biodegradation of the GO-ZnS(Mn) composite was analyzed using Raman spectroscopy after treatment with hMPO for 15 h. The intensity ratio between characteristic D and G bands (I_D_/I_G_) in the 0 h sample was found to be 1.2, while 15 h treated GO-ZnS(Mn) showed the disappearance of such characteristic D and G bands confirming the degradation of the composite by treating with hMPO treatment (Figure 11).

## Conclusions

We developed a biocompatible and biodegradable GO-ZnS(Mn) composite in the aqueous media via *in-situ* growth of ZnS(Mn) QDs on the surface of GO with excellent photoluminescence properties. The GO-ZnS(Mn) composite exhibited excellent colloidal stability and was found to have much higher biocompatibility even at higher concentrations (up to 200 μg/mL). For the first time, the biodegradability of the GO-QDs was also studied by treating with hMPO isolated from the primary immune cells (neutrophils), suggesting GO-ZnS(Mn) composite could be degraded *in vivo* by human neutrophils immune response. Therefore, we believe that this hybrid GO-ZnS(Mn) composite could be helpful for *in vivo* bioimaging and possibly for the synergistic therapy of tumors by delivery of anticancer drugs (chemotherapy) combined with the photothermal therapy (PTT) based on GO.

## Supplementary Material

Supplementary figures.
