# Supplementary material for: Biodegradable Nanocomposite of ZnS(Mn) Quantum Dots Immobilized Graphene Oxide for Bioimaging Applications
Source: Nanotheranostics. 2024 Jan 21;8(2):150–62. doi: 10.7150/ntno.87536 (PMC10845254; doi:10.7150/ntno.87536)
Supplement: Supplementary file 1 — Supplementary figures. [file ntnov08p0150s1.pdf]

Supplementary Information

Biodegradable Nanocomposite of ZnS(Mn) Quantum Dots Immobilized Graphene Oxide for Bioimaging Applications

Pavithra Kurungottu[#a], Midhun Ben Thomas,[#b] Mahesh ML,[a] Prathiksha G,[a] Divya Prakash Gnanadhas,[c,d] Dipshika Chakravortty,[c] Ashok M Raichur,[b]* and Rajendra Kurapati[a*]

[a] School of Chemistry, Indian Institute of Science Education and Research Thiruvananthapuram, Thiruvananthapuram, India

[b]Department of Materials Engineering, Indian Institute of Science, Bangalore, India

[c] Department of Microbiology and Cell Biology, Indian Institute of Science, Bangalore, India

[d] Department of Bioengineering, School of Chemical and Biotechnology, SASTRA Deemed to be University, Thanjavur, Tamilnadu., India

[#] equal contributing authors

[*] Corresponding author: amr@materials.iisc.ernet.in, and rkurapati@iisertvm.ac.in

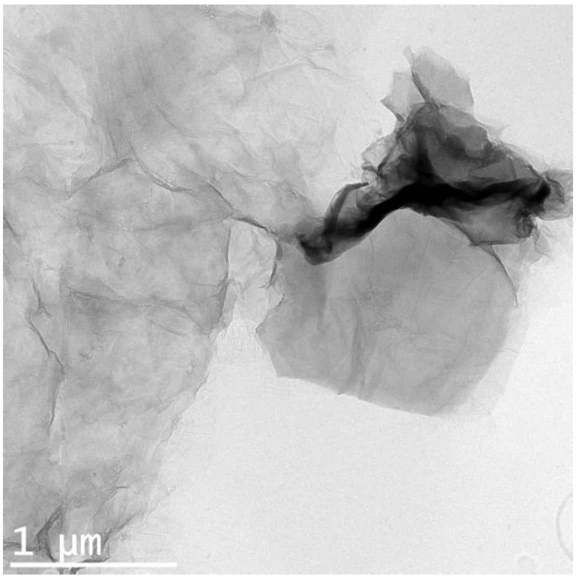

Figure S1. TEM image of exfoliated graphene oxide.

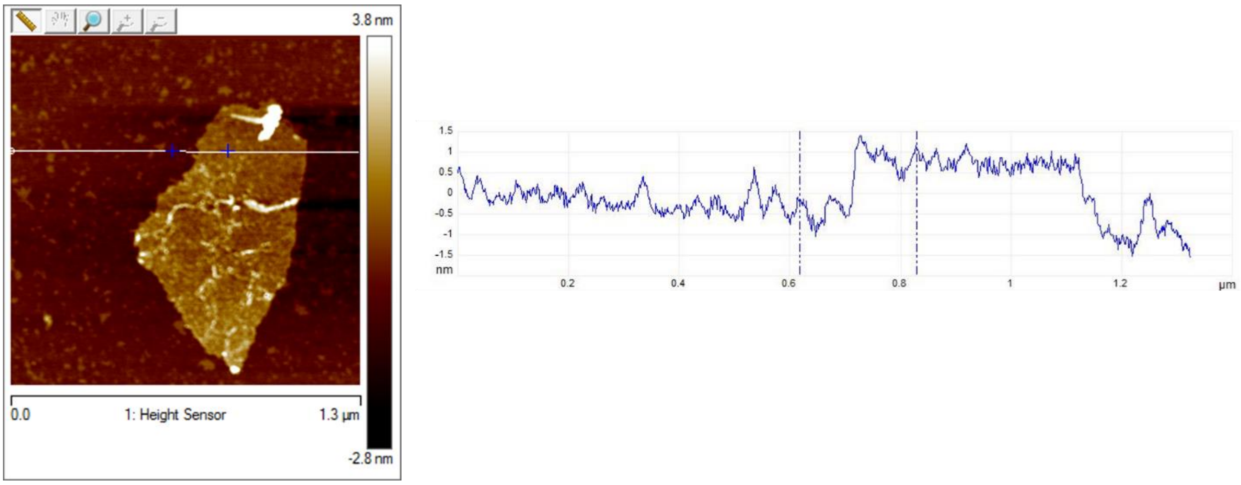

Figure S2. AFM image of exfoliated graphene oxide along with line profile thickness measurement.

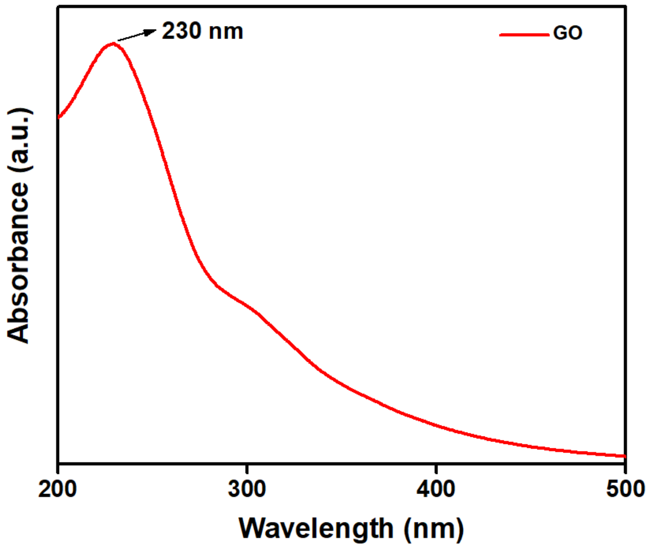

Figure S3. UV-visible spectrum of graphene oxide.

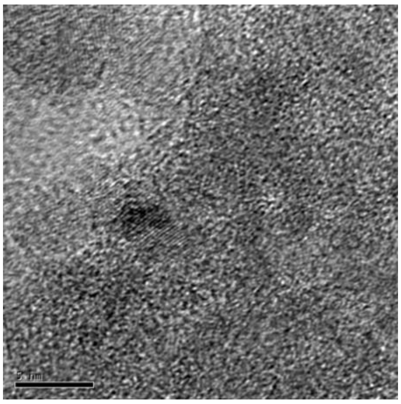

Figure S4. High-resolution TEM image of GO-ZnS(Mn) composite.

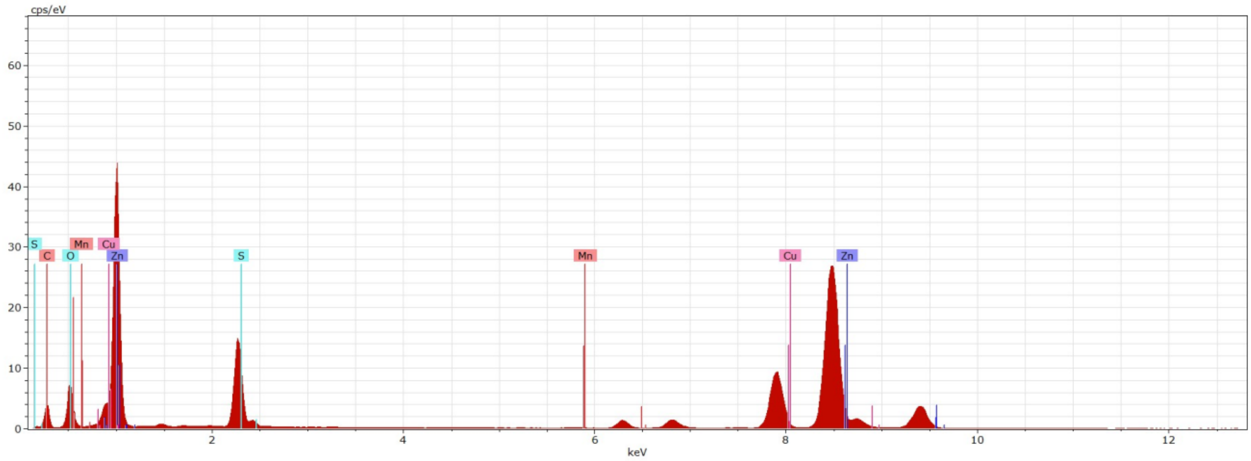

Spectrum: GOZNS 2 737.xls

El	AN	Series	unn. C [wt.%]	norm. C [wt.%]	Atom. C [at.%]	Error (1 Sigma) [wt.%]	K fact.	Z corr.	A corr.	F corr.
C	6	K-series	38.66	38.66	59.32	2.16	1.000	119.210	0.015	1.000
S	16	K-series	23.76	23.76	13.66	1.33	1.000	2.762	0.022	1.000
O	8	K-series	18.76	18.76	21.60	1.06	1.000	42.072	0.015	1.001
Zn	30	K-series	13.13	13.13	3.70	0.58	1.000	0.184	0.311	1.000
Cu	29	K-series	4.17	4.17	1.21	0.21	1.000	0.212	0.269	1.000
Mn	25	K-series	1.52	1.52	0.51	0.10	1.000	0.396	0.119	1.000
		Total:	100.00	100.00	100.00					

Figure S5: EDS spectrum of GO-ZnS(Mn) composite and the elemental composition shown in the table.

Synthesis of CdS Quantum Dots

CdS quantum dots were synthesized using the earlier report[1]. In brief, cadmium acetate dehydrates (0.006 M) solution was added to the freshly prepared sodium sulfide solution (0.06 M). The mixture was stirred at 50 ^{o}C for 15 min, followed by adding 0.4 mL of 2-Mercaptoethanol (capping agent) to regulate the particle size. The gradual appearance of green color confirmed the formation of CdS QDs. The resulting solution was filtered and washed with excess acetone and water.

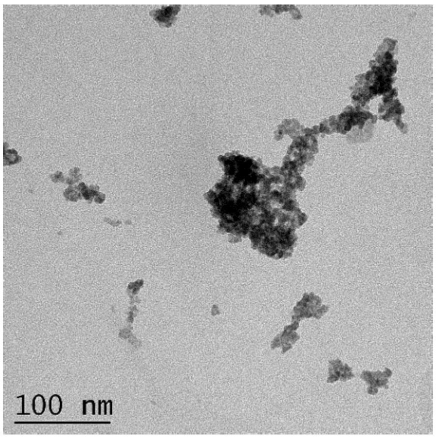

Figure S6. TEM image of CdS QDs

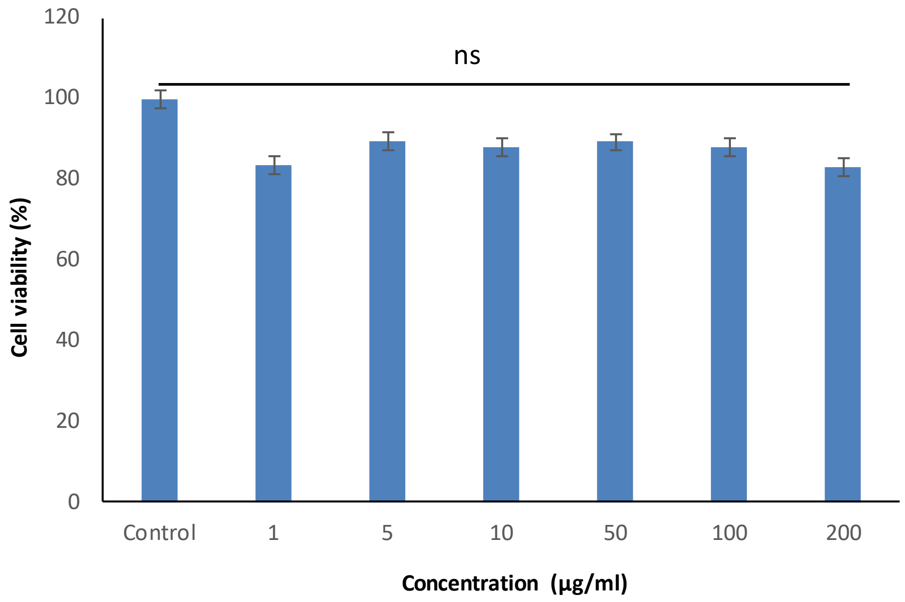

Figure S7. Cell viability assay of CdS QDs in HeLa cells for 48 h (the statistical analyses was performed using t-test in Microsoft Excel).

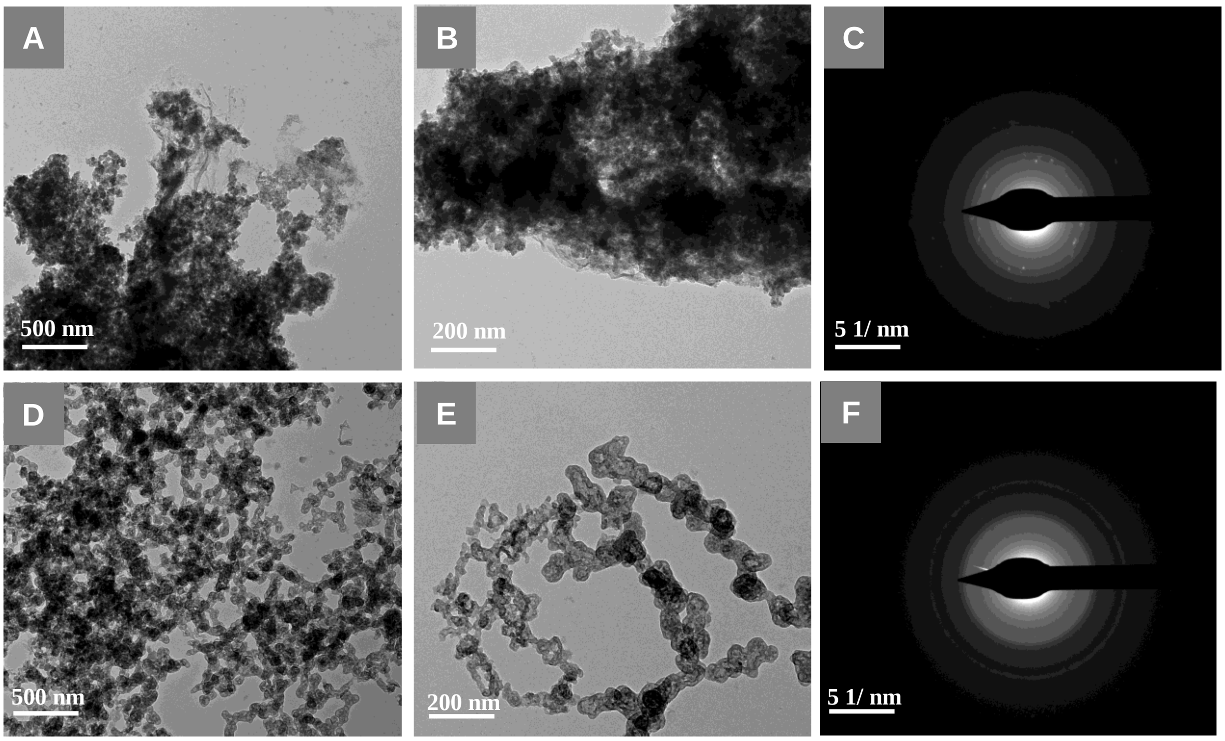

Figure S8: TEM images and SAED pattern of control samples: Images (A-B) correspond to GO-ZnS(Mn) in PBS alone for 15 h at different magnifications; and (C) SAED pattern and (D-F) ZnS(Mn) QDs alone in H_2O_2 for 15 h.

References:

1. Rahul, Singh S, Singh PK, Paul Sharma J, Kakroo S, Sonker R, et al. Encompassing environment synthesis, characterization and photovoltaic utilization of cadmium sulphide quantum dots. Materials Today: Proceedings. 2021; 34: 767-70.
